# Development and Validation of QuEChERS Extraction Coupled with Ultrahigh-Performance Liquid Chromatography–Tandem Mass Spectrometry for the Detection of Nine Macrolides in Fish Products

**DOI:** 10.3390/foods14162768

**Published:** 2025-08-08

**Authors:** Changhua Sun, Yue Ma, Jia Yang, Xubin Lu, Shuai Wang, Xiangfeng Zheng, Zhenquan Yang, Li Xu, Bo Wang

**Affiliations:** 1 Department of Food Technology, College of Biological and Chemical Engineering, Yangzhou Polytechnic University, Yangzhou 225009, China; 101762@yzpc.edu.cn; 2 Key Laboratory of Catering Food Safety and Systematic Monitoring for Jiangsu Province Market Regulation, Yangzhou University, Yangzhou 225127, China; mz120242264@stu.yzu.edu.cn (Y.M.); jiajia82112001@163.com (J.Y.); wshuai608@163.com (S.W.); zxf@yzu.edu.cn (X.Z.); yangzq@yzu.edu.cn (Z.Y.); 3 College of Food Science and Engineering, Yangzhou University, Yangzhou 225127, China; 4 Yangzhou Institute for Food and Drug Control, Yangzhou 225127, China; 5 College of Animal Science and Technology, Yangzhou University, Yangzhou 225009, China; lxb@yzu.edu.cn

**Keywords:** macrolides, quantitative analysis, QuEChERS, UHPLC-MS/MS, aquatic product safety

## Abstract

Veterinary drug residues in aquatic products are often overlooked, yet they pose significant environmental risks and potential threats to human health. In this study, a rapid and sensitive analytical method was developed for the simultaneous determination of nine commonly used macrolide antibiotics in largemouth bass (*Micropterus salmoides*) muscle using ultrahigh-performance liquid chromatography–tandem mass spectrometry (UHPLC-MS/MS). Sample extraction was performed using 80% acetonitrile in water, followed by purification with Cleanert MAS-Q cartridges. Chromatographic separation was achieved on a Waters ACQUITY UPLC BEH C_18_ column (50 mm × 2.1 mm; 1.7 μm), equipped with a Waters VanGuard^TM^ BEH C_18_ guard column (1.7 μm), using a mobile phase consisting of 0.1% formic acid in water and 0.1% formic acid in acetonitrile. Mass spectrometric detection was conducted in positive electrospray ionization mode (ESI^+^) using multiple reaction monitoring (MRM). The method demonstrated excellent linearity in the concentration range of 0.2–30 ng/mL, with determination coefficients (R^2^) exceeding 0.9980 for all analytes. Average recoveries ranged from 89.3% to 108.4%, with intraday and interday relative standard deviations (RSDs) of 2.9–11.6% and 4.1–12.5%, respectively. The limits of detection (LOD) and quantification (LOQ) for largemouth bass muscle were determined to be 0.4 μg/kg and 2.0 μg/kg, respectively. The decision limits (CC_α_) and detection capabilities (CC_β_) ranged from 2.13 to 215.71 μg/kg and 2.22 to 231.42 μg/kg, respectively. The developed method was successfully applied to the quantitative analysis of macrolide residues in 20 largemouth bass samples collected from local markets.

## 1. Introduction

Largemouth bass (*Micropterus salmoides*) is highly favored by consumers due to its firm texture, delicate flavor, minimal bones, and high nutritional value. Aquaculture has become a vital and rapidly expanding industry worldwide. While intensive farming practices have significantly improved economic returns, they also increase the risk of outbreaks and transmission of infectious diseases [1]. To mitigate economic losses associated with such outbreaks, large quantities of antibiotics are frequently administered, both for therapeutic and prophylactic purposes [2]. However, the inappropriate or excessive use of antibiotics can result in environmental contamination, the development of antibiotic-resistant bacteria in aquatic organisms, and potential threats to human health.

**Figure 1:**
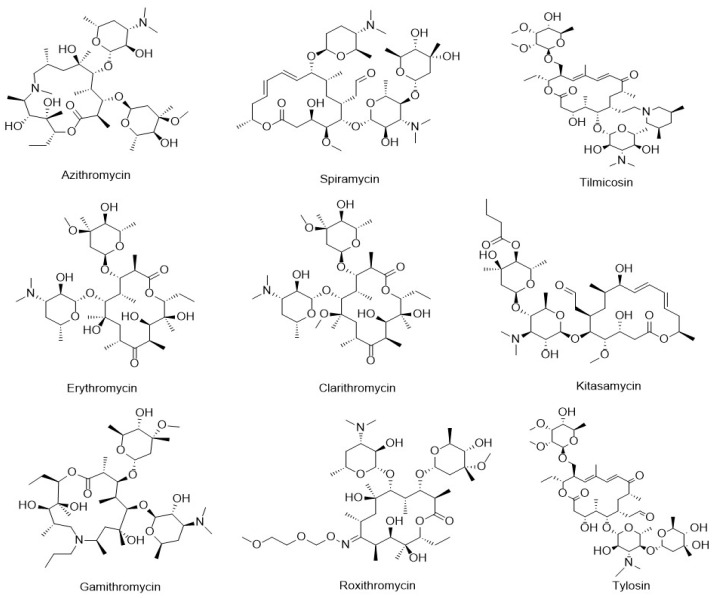
Chemical structures of nine typical MACs.

Macrolides (MACs) are a class of antibiotics produced by *Streptomyces* species, characterized by a macrocyclic lactone ring as the core structure, to which one or more sugar moieties are attached via glycosidic bonds [3]. Common MACs include azithromycin (AZI), spiramycin (SPI), tilmicosin (TIL), erythromycin (ERY), clarithromycin (CLA), kitasamycin (KIT), gamithromycin (GAM), roxithromycin (ROX), and tylosin (TYL), as illustrated in Figure 1. MACs exhibit strong activity against gram-positive bacteria and certain gram-negative bacteria, making them widely used in veterinary medicine and aquaculture [4]. The primary mechanism of action of MACs involves inhibition of bacterial protein synthesis. They exert their antibacterial effect by binding to the 50S subunit of the bacterial ribosome, thereby disrupting peptide chain elongation and translocation, ultimately leading to the cessation of protein production [5,6]. Although the application of these antibiotics has effectively reduced mortality in livestock and aquatic species, the persistence of their residues poses significant risks to human health. Documented adverse effects include hypersensitivity reactions, carcinogenicity, mutagenicity, teratogenicity, and disturbances to the gut microbiota [7,8]. To safeguard public health, many countries have established maximum residue limits (MRLs) for MACs in animal-derived foods, including fish and other aquatic products [9,10,11]. Given the potential hazards associated with antibiotic residues in aquaculture, the development of a rapid, sensitive, and straightforward analytical method for the detection of MAC residues in aquatic products is of critical importance.

Animal-derived and aquatic products are rich in proteins, lipids, vitamins, and minerals, which pose significant challenges for trace-level residue analysis due to matrix interferences. Therefore, sample pretreatment is a critical step prior to instrumental analysis. To date, various sample preparation techniques have been developed and applied for the detection of MAC residues in animal-derived foods, including liquid–liquid extraction (LLE), solid-phase extraction (SPE), matrix solid-phase dispersion (MSPD), accelerated solvent extraction (ASE), dispersive solid-phase extraction (d-SPE), quick, easy, cheap, effective, rugged, and safe (QuEChERS), QuEChERS combined with dispersive liquid–liquid microextraction (DLLME), and molecularly imprinted polymer monolith microextraction (MIPMME) [4,12,13,14,15,16,17,18]. QuEChERS, originally developed based on d-SPE, is a modern sample preparation technique. Its working principle is akin to that of high-performance liquid chromatography (HPLC) and SPE, relying on interactions between the adsorbent and matrix interferences to remove impurities and achieve effective purification [2]. Compared with traditional methods such as LLE and SPE, QuEChERS is considered an environmentally friendly technique, offering the advantages of simplified operation, low solvent consumption, cost efficiency, rapid processing, and suitability for high-throughput analysis [19]. Given the typically low concentrations of antibiotic residues in aquatic matrices, there is a pressing need for rapid, sensitive, and high-recovery sample preparation methods for trace-level analysis. QuEChERS effectively addresses the limitations of traditional techniques, such as complex procedures, high organic solvent consumption, low recovery, and time inefficiency. Therefore, in this study, the QuEChERS method was employed for the determination of trace levels of MAC residues in largemouth bass muscle.

Currently, a variety of analytical techniques are employed for the quantitative determination of MAC residues in animal-derived foods, including thin-layer chromatography (TLC), fourier transform ion cyclotron resonance mass spectrometry, electrochemical assays, colorimetric assays, enzyme-linked immunosorbent assays (ELISAs), fluorescence microscopy, microbial titer analysis, liquid chromatography (LC), gas chromatography-mass spectrometry (GC-MS), and liquid chromatography–tandem mass spectrometry (LC-MS/MS) [20,21,22,23,24,25,26,27,28,29,30,31,32]. Among these, LC-MS/MS stands out due to its short analysis time, high sensitivity, excellent selectivity, good accuracy, and high-throughput screening capability. It has been widely adopted for the monitoring and safety assessment of antibiotic residues in animal-derived foods. In contrast, techniques such as TLC, ELISAs, electrochemical assays, colorimetric assays, fluorescence microscopy, and microbial titer analysis are often limited by lower sensitivity, specificity, and throughput. GC-MS, while effective for certain analytes, is less suitable for antibiotics due to their high polarity and boiling points, and the need for complex derivatization procedures. With advancements in both liquid chromatography and mass spectrometry, ultrahigh-performance liquid chromatography–tandem mass spectrometry (UHPLC-MS/MS) has emerged as a powerful tool, addressing previous limitations such as high solvent consumption, long analysis times, low sensitivity, and limited throughput [33].

Given that several reported methods suffer from drawbacks such as time consumption, complex procedures, poor reproducibility, and low sensitivity and recovery, there is an urgent need to develop a new method to address these issues. Therefore, we developed a QuEChERS-based sample preparation method combined with UHPLC-MS/MS for the simultaneous determination of nine MACs in largemouth bass (*Micropterus salmoides*) muscle. In accordance with European Union (EU) and U.S. Food and Drug Administration (FDA) guidelines [34,35,36], we comprehensively validated the method by specificity, linearity, matrix effect (ME), limit of detection (LOD), limit of quantification (LOQ), decision limit (CC_α_), detection capability (CC_β_), recovery, and precision. Finally, the developed method was applied to 20 largemouth bass samples obtained from local markets to verify its practical applicability and accuracy. This study reduced sample pretreatment and detection time, enhanced the accuracy and sensitivity of the method, and is suitable for the analysis of trace MAC residues in aquatic products.

## 2. Materials and Methods

### 2.1. Chemicals and Reagents

AZI (98.0% purity), SPI (96.9% purity), TIL (93.2% purity), ERY (95.9% purity), CLA (99.1% purity), KIT (98.0% purity), GAM (99.9% purity), ROX (99.9% purity), and TYL (95.8% purity) standards were obtained from Alta Scientific Co., Ltd. (Tianjin, China). Standard stock solutions of AZI, SPI, TIL, ERY, CLA, KIT, ROX, and TYL were prepared at concentrations of 100 μg/mL in methanol, while GAM was prepared at 100 μg/mL in acetonitrile (ACN). HPLC-grade ACN and methanol were purchased from Anpel Laboratory Technologies, Inc. (Shanghai, China), and HPLC-grade formic acid was sourced from Macklin Biochemical Co., Ltd. (Shanghai, China). Cleanert MAS-Q cartridges (containing 50 mg primary secondary amine (PSA), 8 mg Pesticarb (PC), 50 mg C_18_, and 150 mg MgSO_4_) were obtained from Bonna-Agela Technologies (Tianjin, China). Ultrapure water was generated using a Kertone Lab VIP water purification system (Kertone Ltd., Changsha, China). Organic phase nylon syringe filters (13 mm × 0.22 μm) were purchased from Anpel Laboratory Technologies, Inc. (Shanghai, China). Standard working solutions at the required concentrations for method validation were prepared by serial dilution of the 100 μg/mL stock solutions using methanol.

### 2.2. Ethics Statement

This study was approved by and conducted in accordance with the ethical guidelines of the Institutional Animal Care and Use Committee (IACUC) of Yangzhou University. Blank and real sample materials were obtained from local supermarkets and farmers’ markets, respectively. The slaughtering and sampling of largemouth bass were performed by trained personnel following established animal welfare protocols. All necessary measures were taken to minimize the suffering of the fish during handling and sampling procedures.

### 2.3. Sample Preparation

**Figure 2:**
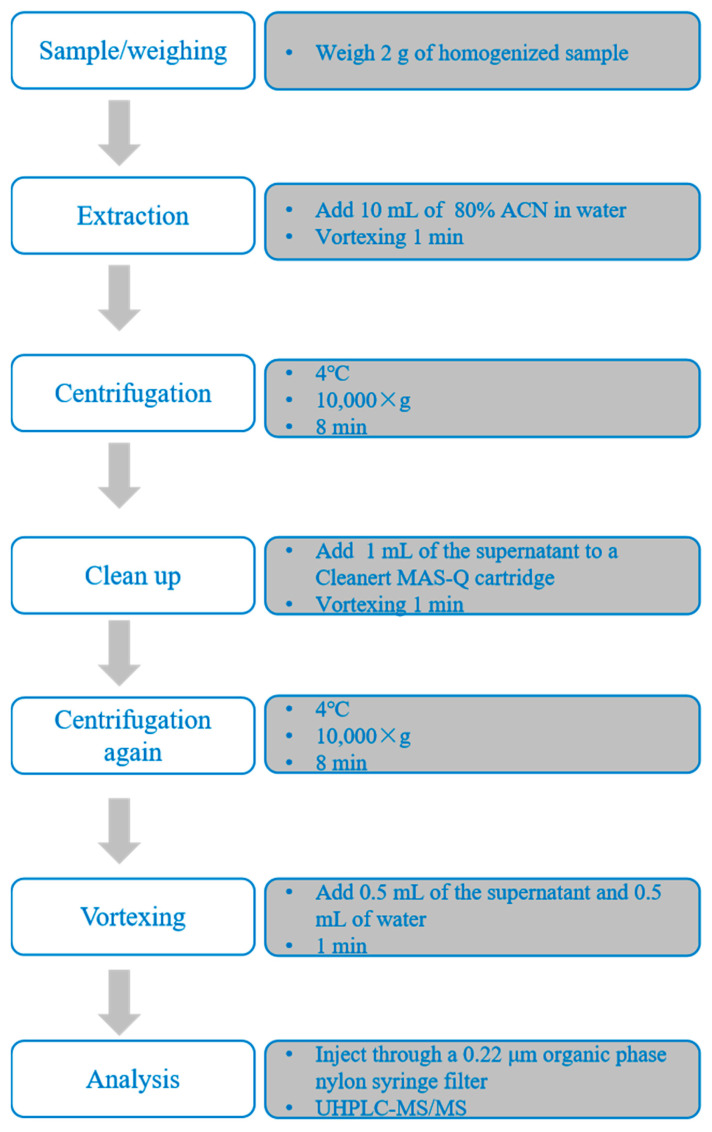
Schematic illustration of the sample preparation procedure for the determination of macrolide residues in largemouth bass muscle.

Sample preparation was carried out following procedures established in our previous studies [19], with the detailed protocol illustrated in Figure 2. The instruments used in sample preparation included the CAT NO. 945066 digital multi-tube vortex mixer (Talboys, Wrens, GA, USA), the 5810R centrifuge (Eppendorf, Hamburg, Germany), and the Vortex Genie 2 vortex mixer (Scientific Industries Inc., Bohemia, NY, USA).

### 2.4. UHPLC-MS/MS Analysis

UHPLC-MS/MS analysis was performed using an ExionLC™ AC UHPLC system coupled with a 6500+ QTRAP mass spectrometer (AB SCIEX, Framingham, MA, USA) equipped with an electrospray ionization (ESI) source. Data acquisition and processing were conducted using Analyst software (version 1.6.3, AB SCIEX). Chromatographic separation was achieved on a Waters ACQUITY UPLC BEH C_18_ column (Waters Corp., Milford, MA, USA, 50 mm × 2.1 mm; 1.7 μm), equipped with a Waters VanGuard™ BEH C_18_ guard column (1.7 μm).

Chromatographic separation was carried out using a binary solvent system consisting of 0.1% formic acid in water (mobile phase A) and 0.1% formic acid in ACN (mobile phase B). The gradient elution program was as follows: 0–1 min, 90:10 (A:B, *v*/*v*); 1–2 min, 10:90 (A:B, *v*/*v*); 2–3 min, 10:90 (A:B, *v*/*v*); 3–4 min, 90:10 (A:B, *v*/*v*); and 4–5 min, 90:10 (A:B, *v*/*v*). The flow rate was set at 0.4 mL/min, the injection volume was 10 μL, and the column temperature was maintained at 40 °C.

Mass spectrometric detection was performed in electrospray ionization positive mode (ESI^+^). The ion spray voltage was set at 5.5 kV, and the source temperature was maintained at 550 °C. Ion source gases 1 and 2 were both set to 50 psi, the curtain gas was set to 35 psi, and the collision gas was set to medium. The dwell time for each transition was 50.0 ms. Quantification was carried out using multiple reaction monitoring (MRM) mode, targeting the protonated molecular ions of each analyte. The optimized precursor ions, product ions, declustering potentials (DP), and collision energies (CE) for all compounds are summarized in Table 1.

### 2.5. Method Validation

The specificity of the method was evaluated by analyzing blank largemouth bass muscle samples to identify any potential interference peaks at the retention times of the target analytes, including AZI, SPI, TIL, ERY, CLA, KIT, GAM, ROX, and TYL. To construct solvent-based calibration curves, a mixed standard working solution was serially diluted with methanol to obtain concentrations of 0.2, 2, 5, 10, 20, and 30 ng/mL. Similarly, matrix-matched calibration curves were prepared by spiking blank largemouth bass muscle matrix extracts with the same concentrations of the mixed standard solution. The matrix effect (ME) was assessed by comparing the slope of the matrix-matched calibration curve to that of the solvent-based calibration curve, using the following formula: ME = slope (matrix-matched)/slope (solvent-based). An ME value greater than 1.2 indicates signal enhancement, while a value less than 0.8 suggests signal suppression. Matrix effects are considered negligible when ME values fall within the range of 0.8–1.2, in which case matrix influence can be ignored [37].

The LOD, LOQ, CC_α_, CC_β_, recovery, and precision of the method were evaluated in accordance with the relevant guidelines outlined in Commission Decision 2002/657/EC, Commission Implementing Regulation (EU) 2021/808, and the FDA [34,35,36]. The definitions and calculation procedures for these validation parameters are consistent with those described in our previously published studies [19].

### 2.6. Statistical Analysis

Experimental data are presented as the mean ± standard deviation (X ± SD). Each treatment was performed in triplicate or with six replicates, as specified. Statistical analysis and data visualization were conducted using GraphPad Prism version 8.2 and SPSS version 16.0 (Statistical Product and Service Solutions). Differences between groups were evaluated using one-way analysis of variance (ANOVA) or independent sample *t*-tests, where appropriate. Post hoc comparisons of means were performed using Tukey’s Honestly Significant Difference (HSD) test. A *p*-value of less than 0.05 was considered statistically significant.

## 3. Results and Discussion

### 3.1. Optimization of the UHPLC-MS/MS Conditions

**Figure 3:**
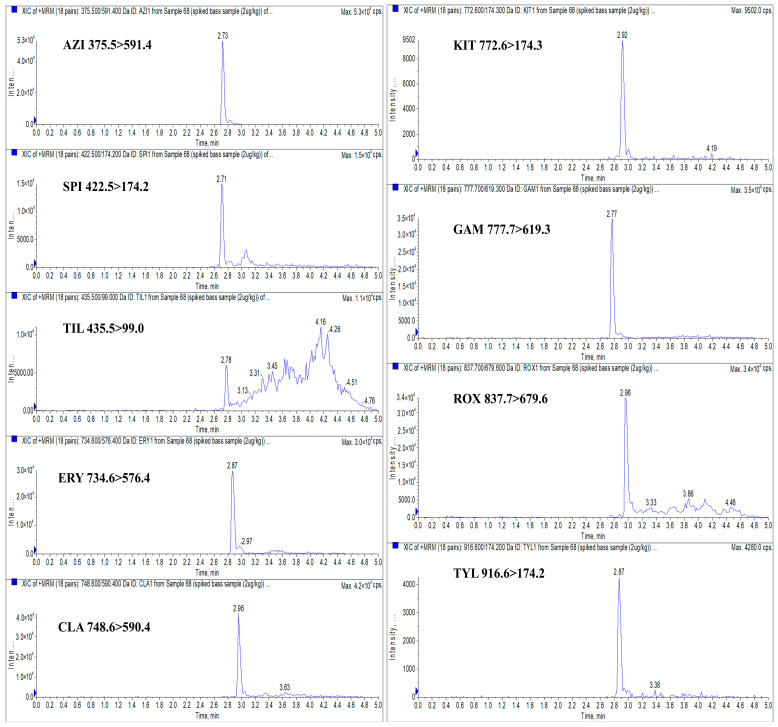
XICs of blank largemouth bass muscle samples spiked with 2 μg/kg AZI, SPI, TIL, ERY, CLA, KIT, GAM, ROX, and TYL. Note: “Inten ..” is represented as “Intensity, cps” in the figure, the following is the same.

Compared with conventional HPLC, UHPLC offers significant advantages, including superior separation efficiency, faster analysis times, higher sensitivity, and reduced consumption of organic solvents. These improvements make UHPLC particularly well-suited for high-throughput trace-level detection of contaminants [38]. The continuous advancement of LC technology has led to a qualitative leap in the rapid screening of chemical residues in food. In this study, UHPLC conditions were adapted from our previously reported method [19]. Chromatographic separation of the target analytes in spiked largemouth bass muscle samples was performed using a Waters ACQUITY UPLC BEH C_18_ column (50 mm × 2.1 mm; 1.7 μm), with a mobile phase consisting of 0.1% formic acid in water and 0.1% formic acid in ACN under gradient elution. The inclusion of 0.1% formic acid not only facilitates protonation of the analytes for enhanced ionization in the mass spectrometer, but also improves chromatographic peak shape and resolution during LC separation [30]. Currently, the column temperature for UHPLC separation of MACs is set at 40 °C, which generally yields good separation results [13,32]. The flow rate is typically optimized based on the gradient elution program and the length of the chromatographic column to ensure separation of target compounds within the elution time. For UHPLC, the flow rate generally ranges from 0.2 to 0.5 mL/min [13,32,39]. Based on the findings from these studies, we ultimately selected a column temperature of 40 °C and a flow rate of 0.4 mL/min. The extracted ion chromatograms (XICs) of blank largemouth bass muscle samples spiked with 2 μg/kg of AZI, SPI, TIL, ERY, CLA, KIT, GAM, ROX, and TYL are presented in Figure 3. Figure 3 demonstrates that the spiked sample exhibited sharp and symmetrical peaks for quantitative ions of all nine macrolides, with no evidence of peak tailing. Due to the high selectivity of the MS/MS procedure, precursor/product ion peaks for the analytes are well distinguished, with retention times of 2.73, 2.71, 2.78, 2.87, 2.96, 2.92, 2.77, 2.96, and 2.87 min, respectively.

**Figure 4:**
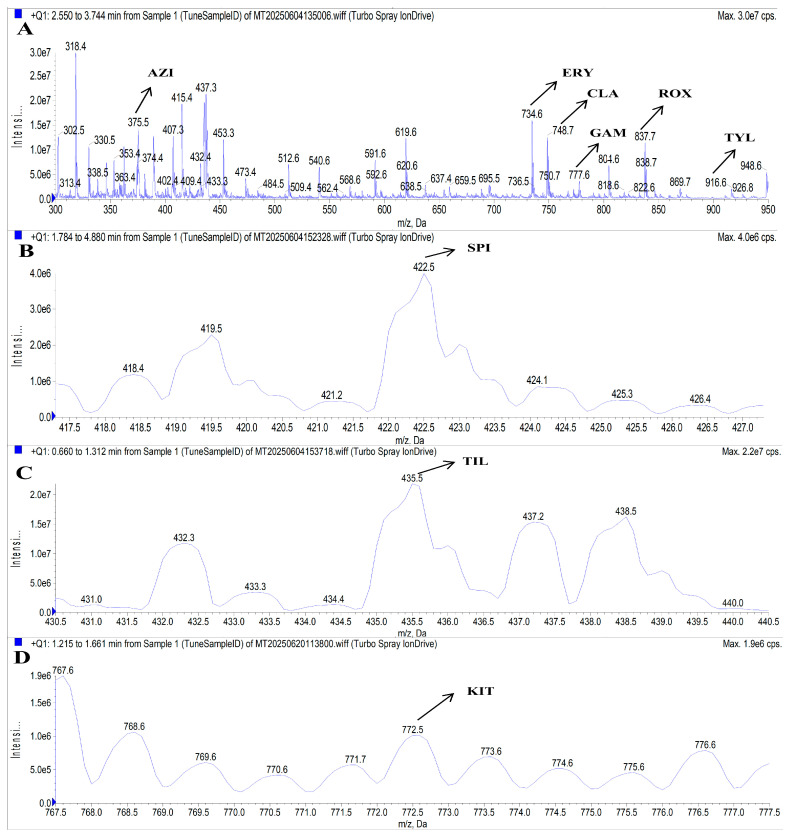
MS spectra of AZI, ERY, CLA, GAM, ROX, and TYL (**A**), SPI (**B**), TIL (**C**), and KIT(**D**). Note: A precursor ion *m*/*z* ± 0.2 was allowed.

To determine the optimal precursor and product ions, as well as DP and CE for the target analytes, a 50 ng/mL mixed standard solution was directly infused into the MS/MS system via a syringe pump method. Given the common structural features of MACs (a macrocyclic lactone ring linked to aminosugar moieties), the ESI^+^ mode was selected for analysis [4]. The nitrogen atoms within MAC molecules readily undergo protonation under ESI^+^ conditions, forming singly, doubly, or triply charged molecular ions [40]. Accordingly, full-scan Q1 MS was performed in the *m*/*z* range of 300–950 in ESI^+^ mode. Preliminary results showed that SPI and KIT exhibited low response intensities, while the signal of TIL was masked by overlapping peaks from other analytes. To enhance precursor ion detection for SPI, KIT, and TIL, the full-scan Q1 MS scanning mode combined with the Center/Width method was employed, with the center set to the molecular weight of the compound and a scan width of 10 Da. Based on signal intensities and MS fragmentation behavior, the selected precursor ions were [M + H]^+^ for ERY, CLA, KIT, GAM, ROX, and TYL, and [M + 2H]^2+^ for AZI, SPI, and TIL. Further comparison of ion responses between [M + H]^+^ and [M + 2H]^2+^ forms confirmed that the doubly charged ions of AZI, SPI, and TIL yielded significantly higher signal intensities than their singly charged counterparts. This is consistent with the findings of Du et al., who reported that the doubly charged ion is the dominant ion for SPI and TIL [32]. As a result, the optimized precursor ions for AZI, SPI, TIL, ERY, CLA, KIT, GAM, ROX, and TYL were *m*/*z* 375.5, 422.5, 435.5, 734.6, 748.6, 772.6, 777.7, 837.7, and 916.6, respectively. These precursor ions are illustrated in Figure 4.

**Figure 5:**
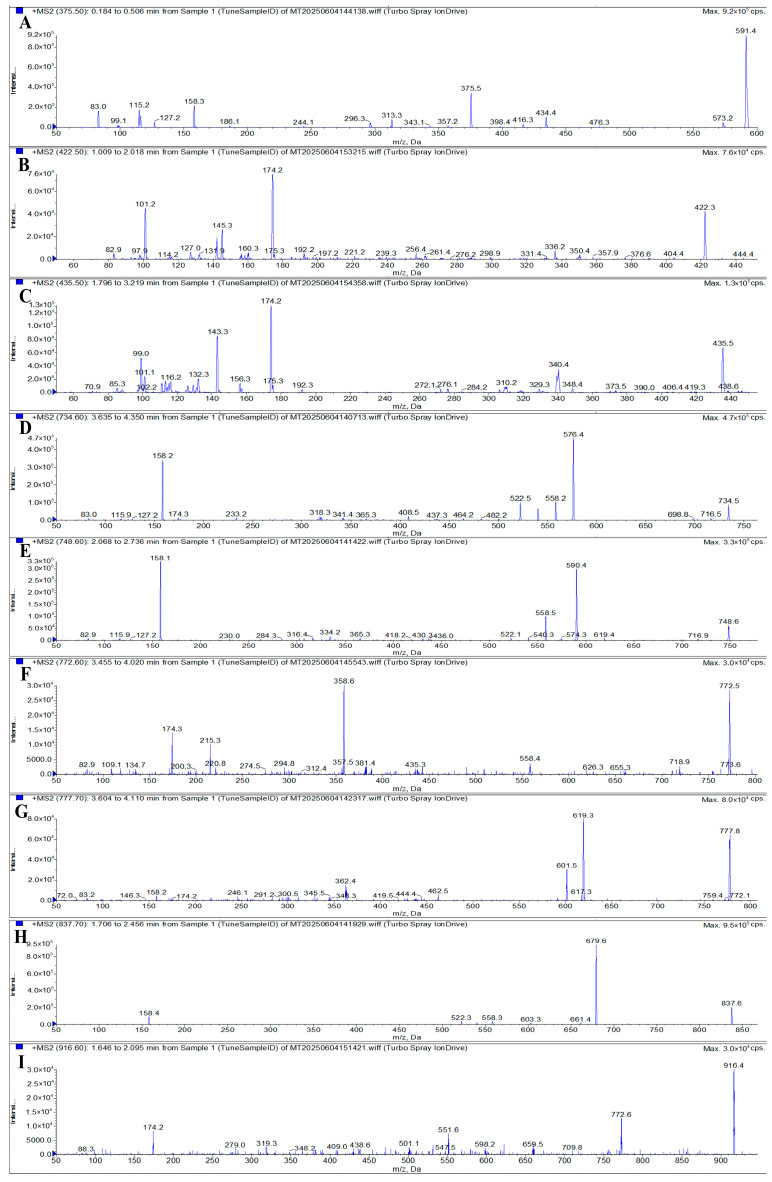
MS/MS spectra of AZI (**A**), SPI (**B**), TIL (**C**), ERY (**D**), CLA (**E**), KIT (**F**), GAM (**G**), ROX (**H**), and TYL (**I**).

To identify suitable product ions for MRM, three to four characteristic fragment ions were initially selected for each analyte based on their fragmentation patterns, as illustrated in Figure 5. DP and CE were then optimized for each transition to maximize signal intensity. Through systematic optimization of UHPLC conditions, MRM transitions, and other MS parameters, two product ions with the highest and most stable response values were selected for each of the nine MACs for use in quantitative and qualitative analysis, respectively. In certain cases, the signal intensities of the selected quantitative and qualitative ion pairs were relatively close. To ensure the robustness and accuracy of the method, both standard mixed working solutions and matrix-matched spiked samples were analyzed to compare signal responses across matrices. Based on a comprehensive evaluation, the most suitable quantifier ions for AZI, SPI, TIL, ERY, CLA, KIT, GAM, ROX, and TYL were determined to be *m*/*z* 591.4, 174.2, 99.0, 576.4, 590.4, 174.3, 619.3, 679.6, and 174.2, respectively. The final optimized MS parameters, including precursor ions, product ions, DP, and CE for both quantifier and qualifier transitions, are summarized in Table 1.

### 3.2. Sample Clean-Up

**Figure 6:**
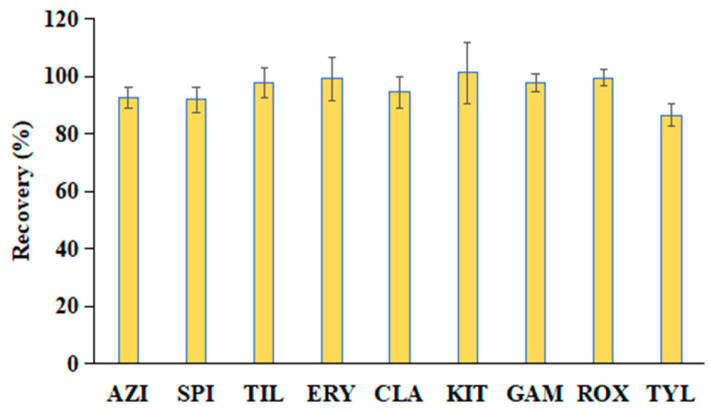
Extraction recoveries of the nine MACs in largemouth bass muscle samples spiked at 200 μg/kg using the proposed QuEChERS method (*n* = 3).

Sample extraction and cleanup are critical steps in the accurate determination of MAC residues in aquatic products. Common pretreatment techniques for MACs include LLE, SPE, d-SPE, and QuEChERS-DLLME [13,18,39,41,42]. However, these methods can be relatively labor-intensive and time-consuming. To address these limitations, a rapid and efficient QuEChERS method was developed in this study, building upon our previous work. The extraction was performed using 80% ACN in water, and sample cleanup was carried out using a commercial Cleanert MAS-Q cartridge (containing 50 mg PSA, 8 mg PC, 50 mg C_18_, and 150 mg MgSO_4_). Due to the hygroscopic nature of MgSO_4_ and its potential to cause solidification of the sorbents during exothermic reactions, thus potentially affecting analyte recovery, the sample was vortexed immediately after adding 1 mL of the supernatant to ensure uniform mixing and minimize loss. The extraction efficiency was evaluated by spiking largemouth bass muscle samples with the nine target MACs (AZI, SPI, TIL, ERY, CLA, KIT, GAM, ROX, and TYL) at a concentration of 200 μg/kg. Three replicates were processed according to the sample preparation workflow shown in Figure 2. In this study, the single-point method was employed to evaluate the extraction recoveries of the target compounds. The formula used is as follows: Extraction recovery = (peak area of spiked sample/peak area of target compound in blank matrix extract) × 100%. As illustrated in Figure 6, the extraction recoveries ranged from 86.6% to 101.3%, with relative standard deviations (RSDs) between 2.8% and 10.7%, demonstrating excellent accuracy and precision. Additionally, each sample could be processed and analyzed in less than 25 min, highlighting the method’s high throughput and operational simplicity compared to traditional approaches. Therefore, the established QuEChERS method can be used to evaluate various methodological parameters.

### 3.3. Matrix Effect (ME) Evaluation

MEs can significantly influence the ionization efficiency of analytes in mass spectrometric analysis and thereby impact quantification accuracy. Sample pretreatment methods can mitigate the influence of MEs through the use of extractants and adsorbents. ACN is effective in precipitating proteins, while adsorbents such as PSA, PC, and C_18_ are commonly used to remove interfering substances. C_18_ removes nonpolar lipids, PC targets certain pigments, and PSA effectively eliminates fatty acids, organic acids, and specific sugars [14,15,18]. In this study, 80% ACN in water was used as the extractant, and a mixture of PSA, PC, and C_18_ was employed as adsorbents to minimize interference from proteins, lipids, and sugars. Additionally, optimizing chromatographic conditions, reducing injection volume, incorporating internal standards, and using matrix-matched calibration can further reduce matrix interferences. The two most straightforward and fundamental techniques to lessen the impact of matrix effects are optimized sample pretreatment and matrix-matching calibration.

To assess this potential interference, the MEs of the nine target MACs were evaluated by comparing the slopes of matrix-matched calibration curves to those of solvent-based calibration curves. As summarized in Table 2, the ME values for AZI, SPI, TIL, ERY, CLA, KIT, GAM, ROX, and TYL in largemouth bass muscle ranged from 0.8 to 1.1. These values fall within the accepted threshold of 0.8–1.2, indicating negligible or minimal matrix effects. Under the optimized sample pretreatment conditions, the external standard method, in conjunction with matrix-matched calibration curves, was deemed appropriate for the quantification and validation of the analytical method.

### 3.4. Method Validation Results

#### 3.4.1. Specificity and Linearity

**Figure 7:**
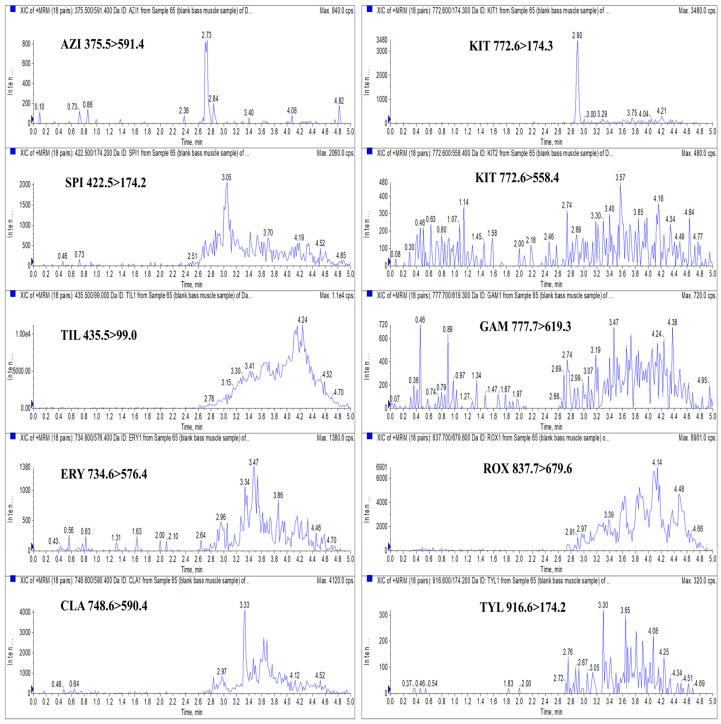
XICs of AZI (375.5 > 591.4), SPI (422.5 > 174.2), TIL (435.5 > 99.0), ERY (734.6 > 576.4), CLA (748.6 > 590.4), KIT (772.6 > 174.3 and 772.6 > 558.4), GAM (777.7 > 619.3), ROX (837.7 > 679.6), and TYL (916.6 > 174.2) in a blank largemouth bass muscle sample.

In this study, blank largemouth bass muscle samples were processed according to the sample preparation procedure described in Section 2.3 and subsequently analyzed using UHPLC-MS/MS. As illustrated in Figure 7, no significant interference peaks were observed at the retention times corresponding to AZI, SPI, TIL, ERY, CLA, GAM, ROX, and TYL, confirming the method’s specificity for these analytes. At the retention time of KIT, a minor solvent or matrix interference peak was detected. However, analysis of both the quantitative and qualitative ion pairs for KIT verified that no KIT residue was present in the blank samples. These findings demonstrate that the method provides sufficient specificity for the accurate identification and quantification of the nine target MACs in largemouth bass muscle and is suitable for further method validation and application.

The linearity of the method was assessed using matrix-matched calibration curves prepared by spiking blank largemouth bass muscle extracts with a mixed standard solution at six concentration levels. Calibration curves were constructed by plotting the peak area of each analyte’s quantifier ion (*y*–axis) against the corresponding concentration (*x*–axis). The results demonstrated excellent linearity over the concentration range of 0.2–30 ng/mL, with determination coefficients (R^2^) of ≥0.9980 for all nine MACs, as summarized in Table 2. These results confirm the method’s suitability for quantitative analysis of trace MAC residues in largemouth bass muscle.

#### 3.4.2. LOD, LOQ, CC_α_, and CC_β_

According to EU regulations, the LOQ is defined as the lowest analyte concentration at which the method yields acceptable recovery (≥70%) and precision (RSD ≤ 20%) values, as stipulated by regulatory guidelines [34]. In this study, the LOQ was determined based on a signal-to-noise (S/N) ratio of 10, following FDA criteria [36]. The S/N ratio was calculated using the peak height relative to the average background noise, measured from the peak-to-peak baseline region adjacent to the analyte signals in the MRM chromatograms. Similarly, the LOD was defined as the lowest concentration at which the analyte could be reliably distinguished from background noise, with an S/N ratio of at least 3. Based on these criteria [34,36], the LOD and LOQ values for nine MACs in largemouth bass muscle were determined to be 0.4 μg/kg and 2.0 μg/kg, respectively. These sensitivity levels are sufficient to meet current regulatory requirements and support routine surveillance monitoring of MAC residues in aquatic products.

In accordance with Commission Implementing Regulation (EU) 2021/808, the CC_α_ and CC_β_ for nine MACs were evaluated using 20 largemouth bass muscle samples spiked at 1 times the MRL or LOQ [35]. According to MRL regulations established by the European Union, the United States, and China, the MRLs for SPI, TIL, ERY, KIT, GAM, and TYL are 200, 50, 200, 200, 150, and 100 μg/kg, respectively [9,10,11]. The calculated CC_α_ and CC_β_ values for all target MACs are presented in Table 2. All results were experimentally validated and confirmed to be within the feasible and detectable range, further supporting the reliability and applicability of the method for regulatory monitoring.

#### 3.4.3. Recovery and Precision

Recovery was determined by analyzing six replicate samples (*n* = 6) fortified at three concentration levels (2, 20, and 200 μg/kg). Notably, after the mixed standard working solution is added to the homogenized blank largemouth bass muscle sample, the mixture should be vortexed for 1 min to ensure thorough contact with the matrix. Precision was evaluated at the same three concentration levels by calculating the relative standard deviation (RSD) for intraday (*n* = 6) and interday (over three consecutive days, *n* = 18) measurements. Both recovery and precision were calculated using matrix-matched calibration curves. As summarized in Table 3, recoveries ranged from 89.3% to 108.4%, with RSDs between 3.1% and 11.7%. Intra-day RSDs ranged from 2.9% to 11.6%, and inter-day RSDs from 4.1% to 12.5%. These results demonstrate that the proposed method exhibits satisfactory sensitivity, accuracy, and precision, and meets the regulatory requirements established by the EU and FDA for the determination of MAC residues in largemouth bass muscle.

### 3.5. Comparison with Other Methods

Currently, the detection of MAC residues in aquatic products primarily relies on LC-MS/MS techniques. LC-MS/MS effectively addresses challenges such as false positives, low throughput, and insufficient sensitivity and accuracy. In this study, we compared various methods for MAC residue detection in terms of recovery, sensitivity, and analysis time. As summarized in Table 4, the QuEChERS-UHPLC-MS/MS method developed herein demonstrated shorter detection time and slightly improved recovery and sensitivity compared with most existing methods. Chen et al. [13] reported a d-SPE-UHPLC-MS/MS method for the determination of 10 MACs in fish, shrimp, crab, and shellfish. Their reported recoveries ranged from 83.1% to 116.6%, with precision (intra-day and interday RSD) values between 3.7% and 13.8%, and LOD and LOQ of 0.25–0.50 μg/kg and 0.5–1.0 μg/kg, respectively. However, the total analytical procedure required more than 53 min to complete. The d-SPE-UHPLC-MS/MS method employed internal standard quantification, whereas our QuEChERS-UHPLC-MS/MS method used external standard calibration for the quantification of 9 MACs in largemouth bass muscle. The proposed method features a simpler sample preparation process, shorter analysis time, and faster throughput. While both methods are suitable for detecting MAC residues in aquatic matrices in terms of recovery, precision, and sensitivity, the QuEChERS-UHPLC-MS/MS method is more advantageous for rapid screening applications due to its simplified workflow and reduced time requirements.

In this study, a QuEChERS-UHPLC–MS/MS method was developed for the simultaneous determination of nine MACs in largemouth bass muscle. The recoveries of the nine MACs ranged from 89.3% to 108.4%, with intra-day and inter-day precision (RSD) below 12.5%. The LOD and LOQ were 0.4 μg/kg and 2.0 μg/kg, respectively. Compared with previously reported methods, this approach offers high sensitivity and accuracy, improved time efficiency, and reduced analytical cost. It provides reliable technical support for the trace analysis of veterinary drug residues in animal-derived foods. Therefore, this method is well-suited for the rapid determination of nine MACs in largemouth bass muscle.

### 3.6. Real Sample Analysis

To evaluate the applicability of the proposed method, 20 largemouth bass samples were collected, individually labeled, and processed using the sample pretreatment procedure described in Section 2.3. The purified extracts were analyzed by UHPLC-MS/MS. Among the nine MACs tested, ERY was detected in only one sample at a concentration of 30.2 μg/kg, while the remaining analytes were not detected in any of the samples. Importantly, none of the samples contained residues exceeding the MRL of 200 μg/kg. These results demonstrate that the QuEChERS-UHPLC-MS/MS method is suitable for the routine monitoring and quantification of MAC residues in largemouth bass muscle.

## 4. Conclusions

In conclusion, this study developed a simple, rapid, and sensitive QuEChERS-UHPLC–MS/MS method for the simultaneous quantification of nine MACs in largemouth bass muscle. A Cleanert MAS-Q cartridge (containing 50 mg PSA, 8 mg PC, 50 mg C_18_, and 150 mg MgSO_4_) was employed during sample pretreatment to effectively eliminate matrix interferences and improve time efficiency. The method utilizes external standard calibration, offering robust performance with satisfactory accuracy and precision. Application of the method to 20 real samples resulted in the detection of ERY in one sample, with all residues remaining below the established MRL. These results confirm the practicality, reliability, and applicability of the proposed method for routine monitoring of MAC residues in largemouth bass muscle. Due to the complexity of animal-derived food matrices, the QuEChERS method still presents certain limitations for the trace analysis of veterinary drug residues in such samples. Future research will focus on developing high-throughput and highly sensitive analytical methods for the simultaneous detection of multiple veterinary drug residues in animal-derived foods.

## Figures and Tables

**Table 1:** The mass spectrum parameters of nine macrolides.

Analyte	Retention Time (min)	Molecular Weight (g/mol)	Molecular Formula	Precursor Ion (*m*/*z*)	Product Ion (*m*/*z*)	Declustering Potential (V)	Collision Energy (eV)
AZI	2.73	749.0	C_38_H_72_N_2_O_12_	375.5	591.4 * 83.0	73	23 22
SPI	2.71	843.1	C_43_H_74_N_2_O_14_	422.5	174.2 * 101.2	68	31 25
TIL	2.78	869.1	C_46_H_80_N_2_O_13_	435.5	99.0 * 174.2	95	26 34
ERY	2.87	733.9	C_37_H_67_NO_13_	734.6	576.4 * 158.2	90	25 37
CLA	2.96	748.0	C_38_H_69_NO_13_	748.6	590.4 * 158.1	95	29 39
KIT	2.92	771.9	C_39_H_65_NO_14_	772.6	174.3 * 558.4	75	43 37
GAM	2.77	777.0	C_40_H_76_N_2_O_12_	777.7	619.3 * 601.5	112	46 49
ROX	2.96	837.0	C_41_H_76_N_2_O_15_	837.7	679.6 * 158.2	110	33 37
TYL	2.87	916.1	C_46_H_77_NO_17_	916.6	174.2 * 772.6	88	46 44

Note: * Quantification ion.

**Table 2:** Regression equations, determination coefficients, decision limits (CC_α_), detection capabilities (CC_β_) and matrix effects (MEs) of nine MACs in largemouth bass muscle.

Analyte	Regression Equation	Determination Coefficient (R^2^)	CC_α_ (μg/kg)	CC_β_ (μg/kg)	ME
AZI	y = 444,404x + 1794.9	0.9999	2.21	2.37	1.0
SPI	y = 153,120x + 4212.1	0.9998	208.45	216.89	1.0
TIL	y = 64,969x − 4049.5	0.9997	57.89	65.78	1.1
ERY	y = 452,604x + 45,786	0.9980	211.73	223.45	1.0
CLA	y = 508,239x + 84,309	0.9998	2.16	2.27	0.8
KIT	y = 92,904x + 7733.7	0.9998	215.71	231.42	0.8
GAM	y = 352,943x + 143,383	0.9981	162.43	174.86	0.9
ROX	y = 478,430x + 13,071	0.9996	2.13	2.22	0.9
TYL	y = 59,211x + 2240.8	0.9998	110.22	120.43	0.8

**Table 3:** Recovery and precision of nine MACs added to the blank largemouth bass muscle samples.

Analyte	Added Level (μg/kg)	Recovery (%) (*n* = 6)	RSD (%) (*n* = 6)	Intraday RSD (%) (*n* = 6)	Interday RSD (%) (*n* = 18)
AZI	2	104.3 ± 5.4	5.2	7.1	9.2
	20	91.1 ± 2.8	3.1	9.0	6.3
	200	93.6 ± 3.8	4.1	6.4	5.7
SPI	2	92.8 ± 4.3	4.6	4.2	4.2
	20	93.7 ± 4.4	4.7	7.9	7.0
	200	94.3 ± 4.4	4.7	6.8	5.4
TIL	2	91.8 ± 6.3	6.9	8.2	7.3
	20	90.3 ± 4.6	5.1	5.9	5.3
	200	95.8 ± 8.1	8.5	8.8	8.8
ERY	2	90.4 ± 5.8	6.4	4.7	5.0
	20	97.7 ± 5.6	5.7	2.9	5.8
	200	100.0 ± 6.0	6.0	4.8	7.5
CLA	2	89.3 ± 6.8	7.6	4.1	6.7
	20	100.7 ± 7.6	7.5	8.0	8.7
	200	94.0 ± 8.1	8.6	9.5	11.2
KIT	2	108.4 ± 5.3	4.9	6.1	5.8
	20	101.8 ± 8.3	8.2	8.5	7.8
	200	107.9 ± 10.3	9.5	9.8	9.0
GAM	2	107.5 ± 9.3	8.7	7.3	11.7
	20	101.2 ± 8.5	8.4	7.7	7.4
	200	96.6 ± 8.6	8.9	7.5	7.8
ROX	2	92.3 ± 5.6	6.1	5.8	7.4
	20	97.3 ± 11.4	11.7	5.9	11.6
	200	97.3 ± 10.8	11.1	11.6	12.5
TYL	2	92.5 ± 4.1	4.4	5.4	6.3
	20	91.3 ± 3.3	3.6	4.1	4.1
	200	89.4 ± 4.0	4.5	7.8	7.1

**Table 4:** Comparison of the proposed method with previously published methods in aquatic products.

Detection Method	Sample Preparation Method	Animal-Derived Food	Analyte	Recovery (%)	LOD (μg/kg)	LOQ (μg/kg)	Detection Time (min)	Ref.
HPLC-MS/MS	SPE	Fish and shrimp	AZI, ERY, and CLA	92.0–99.2	0.053–0.417	0.159–1.251	18	[41]
UHPLC-MS/MS	LLE	Shrimp	SPI, ERY, TIL, TYL, and JOS	74.3–111.1	2.0	5.0	15	[42]
HPLC-MS/MS	QuEChERS-DLLME	Fish	ERY	103–110	0.1	1	7	[18]
UHPLC-MS/MS	d-SPE	Fish, shrimp, crab, and shellfish	AZI, SPI, TIL, ERY, CLA, KIT, ROX, TYL, OLE, and JOS	83.1–116.6	0.25–0.50	0.50–1.00	10	[13]
UHPLC-MS/MS	QuEChERS	Fish	AZI, SPI, TIL, ERY, CLA, KIT, GAM, ROX, and TYL	89.3–108.4	0.4	2.0	5	This study

Abbreviations: azithromycin, AZI; spiramycin, SPI; tilmicosin, TIL; erythromycin, ERY; clarithromycin, CLA; kitasamycin, KIT; gamithromycin, GAM; roxithromycin, ROX; tylosin, TYL; oleandomycin, OLE; josamycin, JOS.

## Data Availability

The original contributions presented in the study are included in the article, further inquiries can be directed to the corresponding authors.

## References

[1] Guidi L.R., Santos F.A., Ribeiro A.C., Fernandes C., Silva L.H., Gloria M.B. (2017). A simple, fast and sensitive screening LC-ESI-MS/MS method for antibiotics in fish. Talanta.

[2] Wang B., Xie K., Lee K. (2021). Veterinary drug residues in animal-derived foods: Sample preparation and analytical methods. Foods.

[3] Hu C., Zhang Y., Zhou Y., Liu Z.F., Meng Q., Feng X.S. (2020). A review of pretreatment and analysis of macrolides in food (Update Since 2010). J. Chromatogr. A.

[4] Tao Y., Yu G., Chen D., Pan Y., Liu Z., Wei H., Peng D., Huang L., Wang Y., Yuan Z. (2012). Determination of 17 macrolide antibiotics and avermectins residues in meat with accelerated solvent extraction by liquid chromatography-tandem mass spectrometry. J. Chromatogr. B.

[5] Kaiser G. (2009). Protein synthesis inhibitors: Macrolides mechanism of action animation. Classification of Agents Pharmamotion.

[6] Chen J., Ying G.G., Deng W.J. (2019). Antibiotic residues in food: Extraction, analysis, and human health concerns. J. Agric. Food Chem..

[7] Beyene T. (2016). Veterinary drug residues in food-animal products: Its risk factors and potential effects on public health. J. Vet. Sci. Technol..

[8] Boobis A., Cerniglia C., Chicoine A., Fattori V., Lipp M., Reuss R., Verger P., Tritscher A. (2017). Characterizing chronic and acute health risks of residues of veterinary drugs in food: Latest methodological developments by the joint FAO/WHO expert committee on food additives. Crit. Rev. Toxicol..

[9] Commission Regulation (EU) (2010). No.37/2010 of 22 December 2009 on pharmacologically active substances and their classification regarding maximum residue limits in foodstuffs of animal origin. Off. J. Eur. Union..

[10] Chinese Ministry of Agriculture and Rural Affairs (2019). National food safety standard-Maximum residue limits for veterinary drugs in foods.

[11] US Food and Drug Administration (2014). CFR-Code of Federal Regulations Title 21 Part 556 Tolerances for Residues of New Animal Drugs in Food.

[12] Shaaban H., Mostafa A. (2023). Simultaneous determination of antibiotics residues in edible fish muscle using eco-friendly SPE-UPLC-MS/MS: Occurrence, human dietary exposure and health risk assessment for consumer safety. Toxicol. Rep..

[13] Chen J., Mei G., Zhang X., Huang D., He P., Xu D. (2024). Dispersive solid-phase extraction and ultra-performance liquid chromatography-tandem mass spectrometry-a rapid and accurate method for detecting 10 macrolide residues in aquatic products. Foods.

[14] Zhou W., Ling Y., Liu T., Zhang Y., Li J., Li H., Wu W., Jiang S., Feng F., Yuan F. (2017). Simultaneous determination of 16 macrolide antibiotics and 4 metabolites in milk by using Quick, Easy, Cheap, Effective, Rugged, and Safe extraction (QuEChERS) and high performance liquid chromatography tandem mass spectrometry. J. Chromatogr. B..

[15] Xu J., Yang M., Wang Y., Yang Y., Tu F., Yi J., Hou J., Lu H., Jiang X., Chen D. (2021). Multiresidue analysis of 15 antibiotics in honey using modified QuEChERS and high performance liquid chromatography-tandem mass spectrometry. J. Food Compos. Anal..

[16] Song X.Q., Zhou T., Li J.F., Su Y.J., Xie J.M., He L.M. (2018). Determination of macrolide antibiotics residues in pork using molecularly imprinted dispersive solid-phase extraction coupled with LC-MS/MS. J. Sep. Sci..

[17] Song X.Q., Zhou T., Zhang J.H., Su Y.J., Zhou H., He L.M. (2019). Preparation and application of molecularly imprinted monolithic extraction column for the selective microextraction of multiple macrolide antibiotics from animal muscles. Polymers.

[18] Campanharo S.C., da Silva A.F.B., Bleuzen A., da Silva J.J.M., de Freitas L.V.P., Assane I.M., Pilarski F., Paschoal J.A.R. (2023). The association of modified QuEChERS and DLLME to offer high analytical detectability to assess residual depletion profile of erythromycin in fish. Food Chem..

[19] Wang B., Liu S., Zhu Y., Zhang H., Xiong D., Guan T., Zheng X., Yang Z., Zhang T., Zhang G. (2024). Determination of erythromycin, clarithromycin and N-desmethyl-erythromycin A residues in pork, beef and lamb based on a simple and fast extraction procedure followed by ultrahigh-performance liquid chromatography with triple quadrupole/linear ion trap mass spectrometry. J. Food Compos. Anal..

[20] Chen Y., Schwack W. (2014). High-performance thin-layer chromatography screening of multi class antibiotics in animal food by bioluminescent bioautography and electrospray ionization mass spectrometry. J. Chromatogr. A.

[21] Sharkawi M.M., Safwat M.T., Abdelaleem E.A., Abdelwahab N.S. (2023). TLC densitometric analysis of triple antibiotic therapy; Erythromycin, Sulfadiazine and Trimethoprim in different edible chicken tissues. J. Liq. Chromatogr. Relat. Technol..

[22] Liu Y.H., Yang Q.X., Chen X.T., Song Y.M., Wu Q.H., Yang Y.Y., He L.P. (2019). Sensitive analysis of trace macrolide antibiotics in complex food samples by ambient mass spectrometry with molecularly imprinted polymer-coated wooden tips. Talanta.

[23] Lai T., Shu H., Tian X., Ren J., Cui X., Bai H., Xiao X.C., Wang Y.D. (2023). Electrochemical sensor based on molecularly imprinted poly-arginine for highly sensitive and selective erythromycin determination. J. Mater. Sci. Mater. Electron..

[24] Zeng L., Liu L.Q., Kuang H., Cui G., Xu C.L. (2019). A paper-based colorimetric assay for rapid detection of four macrolides in milk. Mater. Chem. Front..

[25] Li L., Wang X.Q., Hou R., Wang Y.L., Wang X., Xie C.Q., Chen Y.S., Wu S.M., Peng D.P. (2022). Single-chain variable fragment antibody-based ic-ELISA for rapid detection of macrolides in porcine muscle and computational simulation of its interaction mechanism. Food Control..

[26] Guo X., Liu Y., Dong W., Hu Q., Li Y., Shuang S., Dong C., Cai L., Gong X. (2021). Azithromycin detection in cells and tablets by N,S co-doped carbon quantum dots. Spectrochim. Acta Part A: Mol. Biomol. Spectrosc..

[27] Hamidian K., Amini M., Samadi N. (2018). Consistency evaluation between matrix components ratio and microbiological potency of tylosin major components. DARU J. Pharm. Sci..

[28] Cañadas R., Martínez R.G., González G.P., Hernando P.F. (2022). Development of a molecularly imprinted polymeric membrane for determination of macrolide antibiotics from cow milk. Polymer.

[29] Kumar A., Bhattacharyya A., Shinde R., Dhanshetty M., Elliott C.T., Banerjee K. (2020). Development and validation of a multiresidue method for pesticides and selected veterinary drugs in animal feed using liquid- and gas chromatography with tandem mass spectrometry. J. Chromatogr. A.

[30] Juan C., Moltó J.C., Mañes J., Font G. (2010). Determination of macrolide and lincosamide antibiotics by pressurised liquid extraction and liquid chromatography-tandem mass spectrometry in meat and milk. Food Control.

[31] Jo M.R., Lee H.J., Lee T.S., Park K., Oh E.G., Kim P.H., Lee D.S., Horie M. (2011). Simultaneous determination of macrolide residues in fish and shrimp by liquid chromatography-tandem mass spectrometry. Food Sci. Biotechnol..

[32] Du J., Li X., Tian L., Li J., Wang C., Ye D., Zhao L., Liu S., Xu J., Xia X. (2021). Determination of macrolides in animal tissues and egg by multi-walled carbon nanotube-based dispersive solid-phase extraction and ultra-high performance liquid chromatography–tandem mass spectrometry. Food Chem..

[33] Wang B., Zhu Y., Liu S., Zhang H., Guan T., Xu X., Zheng X., Yang Z., Zhang T., Zhang G. (2024). Quantitative analysis of erythromycin, its major metabolite and clarithromycin in chicken tissues and eggs via QuEChERS extraction coupled with ultrahigh-performance liquid chromatography-tandem mass spectrometry. Food Chem. X.

[34] The European Communities (2002). Commission decision 2002/657/EC of 12 August 2002 implementing council directive 96/23/EC concerning the performance of analytical methods and the interpretation of results. Off. J. Eur. Communities.

[35] European Commission (2021). 2021/808/EC Commission implementing Regulation (EU) 2021/808 of 22 March 2021 on the performance of analytical methods for residues of pharmacologically active substances used in food-producing animals and on the interpretation of results as well as on the methods to be used for sampling and repealing decisions 2002/657/EC and 98/179/EC. Off. J. Eur. Communities.

[36] U.S. Department of Health and Human Services, Food and Drug Administration, Center for Drug Evaluation and Research, Center for Veterinary Medicine (2018). Guidance for Industry: Bioanalytical Method Validation.

[37] Matuszewski B.K., Constanzer M., Chavez-Eng C. (2003). Strategies for the assessment of matrix effect in quantitative bioanalytical methods based on HPLC−MS/MS. Anal. Chem..

[38] Nováková L., Matysová L., Solich P. (2006). Advantages of application of UPLC in pharmaceutical analysis. Talanta.

[39] Griboff J., Carrizo J.C., Bonansea R.I., Valdés M.E., Wunderlin D.A., Amé M.V. (2020). Multiantibiotic residues in commercial fish from Argentina. The presence of mixtures of antibiotics in edible fish, a challenge to health risk assessment. Food Chem..

[40] Wang J. (2009). Analysis of macrolide antibiotics, using liquid chromatography-mass spectrometry, in food, biological and environmental matrices. Mass. Spectrom. Rev..

[41] Pashaei R., Dzingelevičienė R., Abbasi S., Szultka-Młyńska M., Buszewski B. (2022). Determination of 15 human pharmaceutical residues in fish and shrimp tissues by high-performance liquid chromatography-tandem mass spectrometry. Environ. Monit. Assess..

[42] Susakate S., Poapolathep S., Chokejaroenrat C., Tanhan P., Hajslova J., Giorgi M., Saimek K., Zhang Z., Poapolathep A. (2019). Multiclass analysis of antimicrobial drugs in shrimp muscle by ultra high performance liquid chromatography-tandem mass spectrometry. J. Food Drug Anal..

